# Special AT-rich sequence-binding protein-1 participates in the maintenance of breast cancer stem cells through regulation of the Notch signaling pathway and expression of Snail1 and Twist1

**DOI:** 10.3892/mmr.2015.3192

**Published:** 2015-01-13

**Authors:** ZHENGKUI SUN, CHAO ZHANG, XUESEN ZOU, GUIXIANG JIANG, ZONGQUAN XU, WENTING LI, HUI XIE

**Affiliations:** 1 Department of Breast Surgery, Jiangxi Cancer Hospital, Nanchang, Jiangxi 330029, P.R. China; 2 Department of Clinical Medicine, Medical School of Nanchang University, Nanchang, Jiangxi 330029, P.R. China

**Keywords:** breast cancer, SATB1, cancer stem cells, mammosphere, epithelial-mesenchymal transition

## Abstract

The stem cell populations in cancerous tissues and cell lines vary widely and are often associated with aggressive cases of breast cancer. Despite research on the topic, the mechanism underlying the regulation of the breast cancer stem cell (BCSC) population within tumors remains to be fully elucidated. To investigate the function of special AT-rich sequence-binding protein-1 (SATB1) in the maintenance of the BCSC population, SATB1 was overexpressed with lentivirus in MCF-7 cells or knocked down with shRNA-lentivirus in BT-549 cells. The effects of SATB1 overexpression or knockdown on mammosphere formation, the size of the of BCSC population, cell invasion and tumorigenesis were investigated. Activation of the Notch signaling pathway and expression of Snail1 and Twist1 were also examined in the cells. Overexpression of SATB1 in MCF-7 cells was observed to increase mammosphere formation, the size of the BCSC population, cell invasion and tumorigenesis, accompanied by an increase in the activation of Notch signaling and expression levels of Snail1 and Twist1. Conversely, knockdown of SATB1 in BT-549 cells produced the opposite effects. The results indicated that expression of SATB1 may increase the size of the BCSC population via the activation of the Notch signaling pathway and by increasing expression levels of Snail1 and Twist1.

## Introduction

Breast cancer is a notable cause of morbidity and mortality in females, and is associated with a high incidence of recurrence and treatment failure (1). Growing evidence suggests that in patients with breast cancer, tumor metastasis and poor clinical outcome may be due to the evasion of the effects of systemic therapies by a small subset of stem-like cells, termed breast cancer stem cells (BCSCs) (2). In severe combined immunodeficiency (SCID) model mice, BCSCs are identified by the presence of a combination of CD44^+^/CD24^−^, aldehyde dehydrogenase 1 activity, mammosphere formation and tumorigenicity (3,4). The BCSC population varies widely among cancerous tissues and cell lines and is often associated with aggressive types of breast cancer (5). Alterations in critical signaling pathways such as Notch, Wnt/β-catenin and Hedgehog allow stem cells to undergo uncontrolled proliferation (6,7). Notably, previous studies have suggested that the activation of Notch promotes the expansion of BCSCs. In cells with enriched BCSC markers, Notch-1 signaling activity was observed to increase 4-fold while Notch-4 signaling activity increased 8-fold (8,9). In addition, BCSCs are able to undergo epithelial-mesenchymal transition (EMT), a process that is involved in the facilitation of breast cancer progression (10–12). It has been suggested that Snail1 activity is required for EMT initiation, whereas Twist1 serves a role in the maintenance of EMT in mammary epithelial and breast cancer cells (10,13).

Previous studies have suggested that special AT-rich sequence-binding protein-1 (SATB1), which functions as a genome organizer, is crucial in the progression of breast cancer towards metastasis. Deregulation of SATB1 in malignant cells alone, in lieu of multiple successive genomic aberrations, is sufficient to alter the expression of a large number of genes required for the progression of cancer to metastasis (14,15). In the current study, the effects of SATB1 overexpression or knockdown were investigated on the stem cell populations in the breast cancer cell lines MCF-7 or BT-549, respectively, by assessment of *in vitro* mammosphere formation and CD44^+^/CD21^−^ expression, and observation of tumor formation in SCID mice. A previous study demonstrated that the number of mammospheres generated was an indirect measure of mammary stem cell self-renewal; mammosphere size was representative of progenitor cell proliferation; and that the CD44^+^/CD24^−^ population of breast cancer cells display characteristics of stem cells (3). Thus, these factors were investigated in the present study. In order to determine a possible mechanism of SATB1 in maintaining the BCSC population, the expression levels of Notch1, Notch4, Hes1, Snail1 and Twist1 were examined in the MCF-7 and BT-549 cell lines.

## Materials and methods

### Lentiviral construction and cell transfection

All lentiviral constructs were prepared by Shanghai GeneChem Co., Ltd. (Shanghai, China). Lentivirus GV287-SATB1 and Lentivirus GV115-SATB1-shRNA transfection was conducted in accordance with the manufacturer’s instructions (GeneChem, Co., Ltd). The human SATB1 cDNA was subcloned into the GV287 lentiviral vector (http://www.genechem.com.cn/Zaiti.aspx?zt=GV287) and the human SATB1-small hairpin (sh)RNA targeted to SATB1 or negative control (NC)shRNA (GeneChem, Co., Ltd) were subcloned into the GV115 lentiviral vector (http://www.genechem.com.cn/Zaiti.aspx?zt=GV115). Subsequently, the lentivirus vector and packaging plasmid mixes were transfected into HEK293T cells (American Type Culture Collection, Manassas, VA, USA) using Lipofectamine^®^ 2000 (Invitrogen Life Technologies, Carlsbad, CA, USA). Following 48-h transfection, the Dulbecco’s modified Eagle’s medium (DMEM; Gibco, Life Technologies, Grand Island, NY, USA) was harvested and filtered. Subsequent to confirmation via restriction digestion with AgeI restriction enzyme (New England Biolabs, Beverly, MA, USA) and DNA sequencing performed by GeneChem using a pyrosequencing method, large-scale GV287-SATB1, GV115-SATB1-shRNA and GV115-NC-shRNA viruses were produced and used for the transfection into the breast cancer cell lines.

### Cell lines culture and mammosphere assay

The human breast cancer cell lines MCF-7 and BT-549 were obtained from the American Type Culture Collection (Manassas, VA, USA) and maintained in high-glucose DMEM (GE Healthcare Life Sciences, Logan, UT, USA) supplemented with 10% fetal bovine serum (FBS; GE Healthcare Life Sciences) at 37°C in 5% CO_2_. For transfection, the MCF-7 cells were infected with the GV287-SATB1 or the control lentivirus GV287, whereas BT-549 cells were infected with GV115-SATB1-shRNA or lentivirus GV115-NC-shRNA. Following 12~16 h incubation, the viruses were removed and replaced with fresh DMEM. For the mammosphere experiments, single-cell suspensions of the breast cancer cells were plated on ultra-low attachment plates (Corning Inc., Corning, NY, USA) at a density of 1×10^4^ cells/well in DMEM supplemented with 2% (v/v) B-27 (Invitrogen Life Technologies) and 20 ng/ml EGF and bFGF (Peprotech, Inc., Rocky Hill, NJ, USA). Fresh medium was added to the culture every 48 h and images of the resultant non-adherent mammospheres were captured in triplicate using a digital camera (Coolpix 990; Nikon Corp., Tokyo, Japan) on day 10. The diameters of the spheres were measured using Photoshop CS5 (Adobe Systems, Inc., San Jose, CA, USA) and the average sphere sizes in each of the 10 fields were calculated. Quantification of the efficiency of sphere formation involved counting the mammospheres under a CK40 light microscope (Olympus Corp., Tokyo, Japan) at a magnification of ×10 and recording the number of mammospheres/spheres formed in the 96 wells divided by the original number of single cells seeded, expressed as a percentage.

### Flow cytometric analysis

Adherent cells were lifted using 0.25% (v/v) trypsin and washed with phosphate-buffered saline (PBS) (Spectrum Chemical (Shanghai) Co., Ltd, Shanghai, China), while mammosphere cells were collected via centrifugation for 5 min at 300 × g with a XKA-2200 centrifuge (Xiangyi Group, Changsha, China), dissociated using trypsin and washed with PBS. The dissociated cells were resuspended to a final concentration of 5×10^6^ cells/ml in PBS and 5×10^5^ cells (100 μl) were incubated with 0.5~1.0 μg phycoerythrin (PE)-conjugated mouse anti-human/mouse CD44 (12-0441) and allophycocyanin (APC)-conjugated mouse anti-human CD24 antibodies (17-0247) or isotype matched control antibodies (1:200; BD Biosciences, Heidelberg, Germany) at 4°C for 60 min. The cells were then washed twice with ice-cold PBS and collected by centrifugation for 5 min at 300 × g for flow cytometric analysis using the FACSAria II (BD Biosciences) with FACSDiva software (BD Biosciences) indicating APC (CD24) fluorescence on the x-axis and PE (CD44) fluorescence on the y-axis.

### Invasion assays

A Transwell system (24 wells; 8 μm pore size; BD Biosciences) coated with 2 mg/ml basement membrane Matrigel (BD Biosciences) was used for the *in vitro* invasion assays. A total of 1×10^5^ cells were suspended in serum-free DMEM (Gibco) in the upper chamber of each well, while the lower chamber of each well was filled with 750 μl DMEM supplemented with 10% FBS. Subsequent to suspension for 24 h, the filters were fixed with methanol and stained with 0.1% crystal violet (Spectrum Chemical (Shanghai) Co., Ltd). The number of cells in at least five randomly selected microscope fields (the filter was divided into 16 microscopic fields and five were selected using a random number method) were then counted and underwent statistical analysis.

### Implantation of cells in SCID model mice

MCF-7 cells infected with GV287 or GV287-SATB1 lentivirus, or BT-549 cells infected with GV115-SATB1-shRNA or GV115-NC-shRNA lentivirus, were washed in PBS and then injected into the mammary fat pad of 5-week-old female SCID mice anesthetized with pentobarbital sodium (Sigma-Aldrich) administered via intraperitoneal injection (40 mg/kg). To test the success rates of engraftment, 10 mice of each group were respectively injected with 10^3^, 10^4^ and 10^5^ cells, and to test engraftment size, 6 mice of each group were injected with 10^6^ cells. The mice (20–30 g), obtained from SLRC Laboratory Animals (Shanghai, China) were maintained in laminar flow rooms under constant temperature and humidity and received estradiol supplementation (0.4 mg/kg; Novo Nordisk, Copenhagen, Denmark) every week subsequent to cell injection. Mice were inspected for tumor appearance daily by observation and palpation for 12 weeks subsequent to cell injection. Tumor volumes were calculated using the formula V=L(W^2^)/2, in which L indicates length and W indicates tumor width. At the conclusion of the experiments, mice were sacrificed by cervical dislocation and the presence of each tumor nodule was confirmed by necropsy. Experimental protocols were approved by the Ethics Committee for Animal Experimentation of Jiangxi Cancer Hospital (Nanchang, China).

### Immunoblotting

Cells and tumor tissues were lysed in a laemmli buffer (Bio Rad Laboratories, Inc., Hercules, CA, USA), boiled and loaded onto SDS (Thermo Fisher Scientific, Rockford, IL, USA)-polyacrylamide gels (Energy Chemical, Shanghai, China). Following electrophoresis (Bio Rad Laboratories, Inc.), proteins were transferred onto nitrocellulose membranes (GenScript USA Inc., Piscataway, NJ, USA) using Trans-Blot SD Semi-Dry Electrophoretic Transfer Cell (Bio-Rad Laboratories, Inc.). Blots were incubated in Tris-buffered saline (TBS) (Spectrum Chemical (Shanghai) Co., Ltd) blocking buffer containing 2% milk for 1~2 h at room temperature and then with the mouse monoclonal anti-human antibodies against Notch1 (sc-373891), Hes1 (sc-166410), Snail1 (sc-271977) and Twist1 (sc-81417), as well as rabbit anti-human polyclonal antibodies against SATB1 (sc-28676) and Notch4 (sc-5594) (Santa Cruz Biotechnology, Inc., Santa Cruz, CA, USA) diluted 1:200 in TBS with Tween (TBST; containing 0.1% Tween-20 and 2% bovine serum albumin; Spectrum Chemical (Shanghai) Co., Ltd) overnight at 4°C. Subsequently, blots were washed and incubated with the appropriate secondary antibodies (goat polyclonal anti-mouse IgG, sc-2005, and goat polyclonal anti-rabbit IgG, sc-2004; Santa Cruz Biotechnology Inc.) in TBST at a 1:100–1:200 dilution ratio and detected using the BeyoECL Plus Western Blotting Detection System (Beyotime, Haimen, China), in accordance with the manufacturer’s instructions.

### Histology and immunohistochemistry

Tumor tissues were fixed in 10% neutral buffered formalin (pH 7.4; Spectrum Chemical (Shanghai) Co., Ltd), embedded in paraffin (ApexBio Technology LLC, Houston, TX, USA), cut into 5-μm sections using a CUT6062 automatic paraffin slicing machine (SLEE Medical GmbH, Mainz, Germany) and stained with hematoxylin and eosin (Boster Biological Engineering Co., Ltd, Wuhan, China). For immunostaining, the sections were deparaffinized, rehydrated and stained with the anti-Ki67 antibody (Santa Cruz Biotechnology, Inc.) and VECTASTAIN Elite ABC Kit (Vector Laboratories, Burlingame, CA, USA) according to the manufacturer’s instructions. For each slide examined, 1,000 cells were counted from 6 fields (randomly selected from 16 microscopic fields) at ×20 magnification, and the percentage of Ki67-positive cells was calculated from the number of total cells. The number of Ki67-positive tumor cells in 100 tumor cells determined the Ki67 proliferation index.

### Statistical analysis

Statistical analysis was performed using SPSS, version 19.0 (IBM SPSS, Armonk, NY, USA). Fisher’s exact test was used to determine associations between SATB1 expression and the success rates of engraftment. The differences in the means of the groups were analyzed with one-way analysis of variance or independent-samples t-test. P<0.05 was considered to indicate a statistically significant difference.

## Results

### Knockdown or overexpression of SATB1 reduces or increases the capacity for mammosphere formation in breast cancer cells, respectively

**Figure 1:**
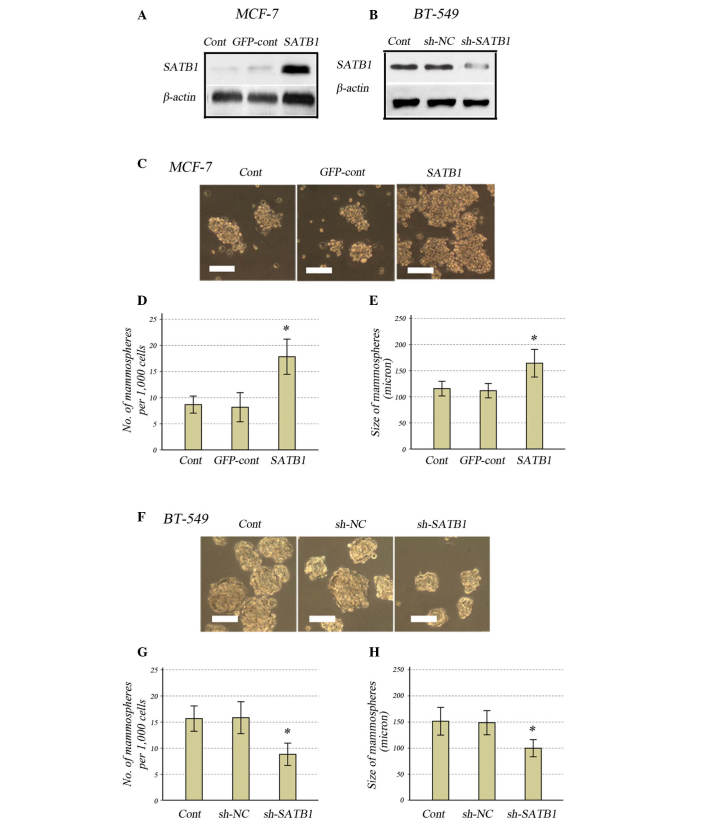
Effects of SATB1 expression on mammosphere formation in human breast cancer cell lines. Western blot analysis for SATB1 expression in (A) MCF-7 and (B) BT-549 cells. (C) Light microscope images of the MCF-7 cells (magnification, ×200; scale bar, 10 μm). (D) Number of and (E) size of mammospheres in SATB1-expressing MCF-7 cells compared with the controls. (F) Light microscope images of the BT-549 cells (magnification, ×200; scale bar, 10 μm). (G) Number of and (H) size of mammospheres in SATB1-knockdown BT-549 cells compared with the controls. All data are presented as the mean ± standard deviation of two independent experiments in triplicate, ^*^P<0.01 vs. controls. SATB1, special AT-rich sequence-binding protein-1; cont, control; GFP, green fluorescent protein; sh, small hairpin; NC, negative control.

To examine the role of SATB1 in maintaining the BCSC population, the lentivirus GV287-SATB1 was selected to overexpress SATB1 in MCF-7 breast cancer cells, while the shRNA-lentivirus GV115-SATB1-shRNA was selected to knockdown SATB1 in BT-549 cells. SATB1 overexpression or knockdown was confirmed by western blotting (Fig. 1A and B). When the MCF-7 cells transfected with GV287-SATB1 were cultured, the size and number of mammospheres significantly increased compared with the controls (Fig. 1C–E). In BT-549 cells transfected with GV115-SATB1-shRNA, the size and number of mammospheres was significantly reduced compared with the controls (Fig. 1F–H).

### Effect of SATB1 expression on the CD44^+^/CD24^−^ population and tumor invasiveness in breast cancer cells

**Figure 2:**
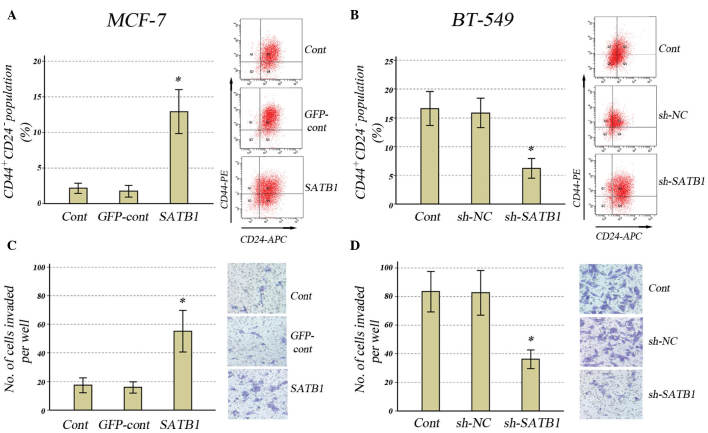
Effects of SATB1 expression on the CD44^+^/CD24^−^ population and invasive properties of human breast cancer cell lines. Populations of CD44^+^/CD24^−^ in (A) MCF-7 cells with SATB1 overexpression and (B) BT-549 cells with SATB1 knockdown, with representative images from the CD44/CD24 flow cytometric analysis. Invasiveness in (C) MCF-7 cells with SATB1 overexpression and (D) BT-549 cells with SATB1 knockdown, and representative images taken from the invasion assays (magnification, ×20). All data are presented as the mean ± standard deviation of two independent experiments in triplicate; ^*^P<0.001 vs. controls. SATB1, special AT-rich sequence-binding protein-1; cont, control; GFP, green fluorescent protein; sh, small hairpin; NC, negative control.

In order to further determine the effects of SATB1 expression on the cancer stem cell population, the effects of SATB1 overexpression in MCF-7 cells and SATB1 knockdown in BT-549 cells on CD44^+^/CD24^−^ populations were investigated. In MCF-7 cells transfected with GV287-SATB1, SATB1 overexpression resulted in a significant increase in the CD44^+^/CD24^−^ population (Fig. 2A). In BT-549 cells transfected with GV115-SATB1-shRNA, downregulation of SATB1 expression resulted in a significant reduction in the CD44^+^/CD24^−^ population (Fig. 2B).

It has previously been suggested that tumor invasion and metastasis may be mediated by the cancer stem cell population (16,17), thus, the Matrigel-invading ability of MCF-7 cells infected with GV287-SATB1 or BT-549 cells infected with GV115-SATB1-shRNA was investigated. Overexpression of SATB1 resulted in a significant increase in invasiveness compared with control groups in the MCF-7 cells (Fig. 2C), whilst downregulation of SATB1 resulted in a significant reduction in invasiveness compared with control groups in BT-549 cells (Fig. 2D).

### Knockdown or overexpression of SATB1 reduces or increases Notch1, Notch4, Hes1, Snail1 and Twist1 expression levels, respectively

**Figure 3:**
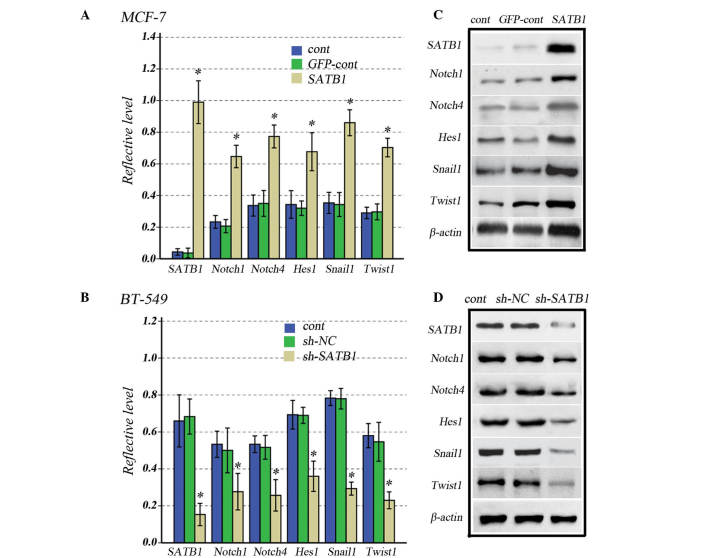
Effects of increased or reduced SATB1 expression on Notch1, Notch4, Hes1, Snail1 and Twist1 expression levels in human breast cancer cells. (A) Graph and (B) representative western blot of SATB1, Notch1, Notch4, Hes1, Snail1 and Twist1 expression levels in SATB1-overexpressing and control MCF-7 cells. (C) Graph and (D) representative western blot of SATB1, Notch1, Notch4, Hes1, Snail1 and Twist1 expression levels in SATB1-knockdown and control BT-549 cells. All data are presented as the mean ± standard deviation of experiments in triplicate; ^*^P<0.001 vs. controls. SATB1, special AT-rich sequence-binding protein-1; cont, control; GFP, green fluorescent protein; sh, small hairpin; NC, negative control.

To determine whether SATB1 affects Notch signaling pathways, the expression levels of genes involved in stem cell behavior, including Notch1, Notch4, Hes1, Snail1 and Twist1, were investigated in MCF-7 and BT-549 cells transfected with GV287-SATB1 and GV115-SATB1-shRNA, respectively. Overexpression of SATB1 significantly increased the expression levels of Notch1, Notch4, Hes1, Snail1 and Twist1 compared with control groups in MCF-7 cells (Fig. 3A), which can be observed by the results of the western blot analysis (Fig. 3B). Conversely, the downregulation of SATB1 reduced the expression of these genes compared with control groups in BT-549 cells (Fig. 3C), and can be observed by the results of the western blot analysis (Fig. 3D).

This suggests that SATB1 overexpression increases the size of the stem cell pool, and also activates Notch signaling pathways, increasing the expression levels of Snail1 and Twist1, which drive EMT in breast cancer cells.

### Effects of SATB1 expression on stem cell population, tumorigenicity and Notch1, Notch4, Hes1, Snail1 and Twist1 expression levels in breast cancer cells in vivo

**Figure 4:**
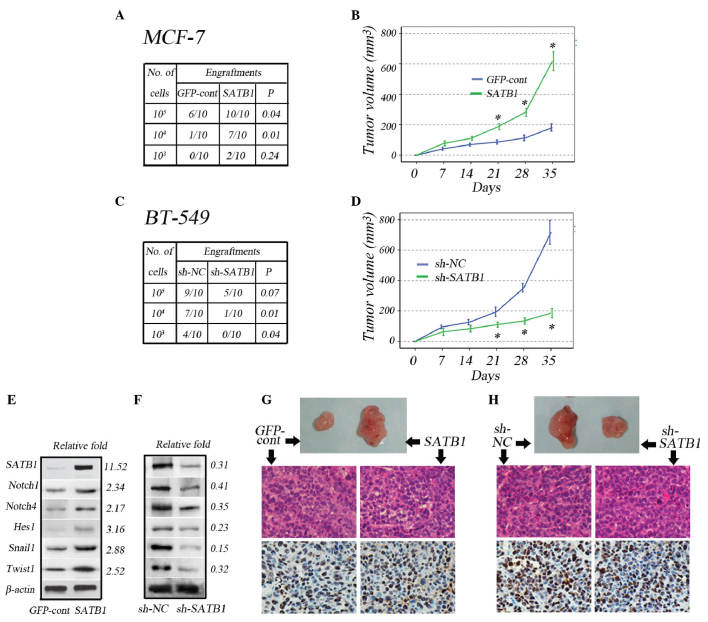
Effects of SATB1 expression on tumorigenicity and expression levels of Notch1, Notch4, Hes1, Snail1 and Twist1 *in vivo.* (A) The number of successful tumor engraftments correlated with the number of MCF-7-GFP-cont and MCF-7-SATB1 cells injected during serial dilution. (B) Tumor growth size subsequent to injection of 1×10^6^ MCF-7-GFP-cont and MCF-7-SATB1 cells. (C) The number of successful tumor engraftments correlated with the number of BT-549-sh-NC and BT-549-sh-SATB1 cells injected during serial dilution. (D) Tumor growth size subsequent to injection of 1×10^6^ BT-549-sh-NC and BT-549-sh-SATB1 cells. Representative immunoblot analysis and expression fold-change of SATB1, Notch1, Notch4, Hes1, Snail1 and Twist1 in (E) MCF-7-GFP-cont and MCF-7-SATB1-derived and (F) BT-549-sh-NC and BT-549-sh-SATB1-derived tumors relative to that of the control group. Representative images of xenografts, hematoxylin and eosin staining and immunohistochemical staining for Ki-67 xenografts, derived from injection of (G) 10^6^ MCF-7-GFP-cont and MCF-7-SATB1 cells (magnification, ×10) and (H) 10^6^ BT-549-sh-cont and BT-549-sh-SATB1 cells (magnification, ×10). Quantitative data are presented as the mean ± standard deviation for six tumors in each group; ^*^P<0.001 vs. controls. SATB1, special AT-rich sequence-binding protein-1; GFP, green fluorescent protein; cont, control; sh, small hairpin; NC, negative control; relative fold, expression fold-change relative to the control group.

A subcutaneous serial dilution transplantation experiment was performed in female SCID mice using MCF-7 and BT-549 cells transfected with GV287-SATB1 and GV115-SATB1-shRNA, respectively. A notable observation was that following the subcutaneous implantation of 1×10^4^ and 1×10^5^ MCF-7 cells, SATB1 overexpression in the cells significantly increased the success of engraftments (Fig. 4A), and subsequent to the implantation of 1×10^6^ MCF-7 cells, SATB1 overexpression significantly increased tumor size after 21 days (Fig. 4B). Following the subcutaneous implantation of 1×10^3^ and 1×10^4^ BT-549 cells, SATB1 depletion in the cells significantly reduced the success of engraftments (Fig. 4C), while subsequent to the subcutaneous implantation of 1×10^6^ BT-549 cells, SATB1 knockdown significantly reduced tumor size after 21 days (Fig. 4D).

In agreement with the *in vitro* results, western blot analysis of the tumors revealed that *in vivo* overexpression of SATB1 in MCF-7 cells increased the expression levels of Notch1, Notch4, Hes1, Snail1 and Twist1 (Fig. 4E), whereas knockdown of SATB1 in BT-549 cells reduced the expression levels (Fig. 4F). Immunohistochemical analyses of the tumors revealed that the Ki67 proliferation index was comparable between MCF-7-GFP and MCF-7-SATB1 tumors (Fig. 4G), despite an increase in tumor size. In addition, the Ki67 proliferation index was comparable between BT-549-GFP and BT-549-sh-SATB1 tumors, despite a reduction in tumor size (Fig. 4H).

## Discussion

Although SATB1 overexpression has been previously demonstrated to increase breast cancer invasion and metastasis, its effect on the stem cell population remains to be fully elucidated. In the current study, SATB1 was identified to expand the cancer stem cell population, accompanied by the activation of the Notch signaling pathway, which promotes cancer stem cell self-renewal and the expression of the Snail1 and Twist1 genes that drive EMT.

Overexpression of SATB1 in the breast cancer cell line MCF-7 was demonstrated to increase the population, which was measured by mammosphere formation and CD44^+^/CD24^−^ expression *in vitro* and formation of tumors in SCID model mice (*in vivo).* Downregulation of SATB1 in the breast cancer cell line BT-549 resulted in the opposite result. Previous studies have demonstrated that the subpopulation of breast cancer cells exhibit increased invasive properties (17). SATB1 overexpression increased the invasiveness of MCF-7 cells, and SATB1 knockdown reduced the invasiveness of BT-549 cells. These results suggest that SATB1 expression in breast cancer may increase the BCSC population, resulting in tumor progression.

Aberrant Notch signaling has been identified to be associated with the development and progression of breast cancer (18,19); the Notch signaling pathway maintains the stemness of BCSCs (20). Previous studies have demonstrated that Notch signaling activity increased in BCSCs, as demonstrated by increased expression of the Notch receptors Notch1 and Notch4, in addition to Hes-1, an immediate downstream target of Notch signaling (8). In the current study, SATB1 overexpression in MCF-7 cells was demonstrated to significantly increase Notch1, Notch4 and Hes1 expression, whilst its knockdown in BT-549 cells resulted in the opposite effect. These data suggest that SATB1 mediates the BCSC population via the Notch signaling pathway.

Additionally, it has been hypothesized that the progression of the majority of carcinomas towards malignancy is associated with EMT, during which breast cancer cells transition to acquire stem cell-like properties (21,22). In cancer EMT, signals emanate via factors, including transforming growth factor-β, hepatocyte growth factor, epidermal growth factor and hypoxia (23). The signals then converge upon a limited set of transcriptional repressors, including Snail1, Slug, ZEB1/2 and Twist1/2 (24,25). One study suggested that Snail1 activity is required for EMT initiation, whereas Twist1 is involved in the maintenance of EMT during human breast cancer progression towards metastasis (13). In the current study, it was observed that Snail1 and Twist1 were upregulated in MCF-7 cells with SATB1 overexpression and inhibited in BT-549 cells with SATB1 knockdown. Therefore, in the present study, SATB1 expression was established to affect the number of BCSCs, in addition to their ability to propagate in conditions sustaining the undifferentiated cell state.

In conclusion, the current study suggests that the expression of SATB1 may increase the size of the BCSC population via the activation of Notch signaling, which is required for maintaining the stemness of BCSCs and increasing the expression level of Snail1 and Twist1, which are required for EMT.
